# USE OF SENSOR TECHNOLOGY TO MAP THE SOCIAL NETWORKS OF PEOPLE LIVING WITH DEMENTIA: A FEASIBILITY STUDY

**DOI:** 10.1093/geroni/igz038.281

**Published:** 2019-11-08

**Authors:** Kayla Wright-Freeman, Sijia Wei, Eleanor McConnell, Kevin Caves, Leighanne Davis, Adrienne Hawkes, Sarah Moninger, Kirsten N Corazzini

**Affiliations:** 1 Duke University, Durham, North Carolina, United States; 2 Duke, Hillsborough, North Carolina, United States

## Abstract

For older adults living with dementia, social network quality influences health outcomes. However, current social network measurement methods are time consuming and mentally draining for people living with dementia. This study aimed to accurately measure social networks using sensor technology. Bluetooth and radio-frequency identification (RFID) sensors were used to collect social network data in a simulation of a falling nursing home resident living with dementia. Participants wore sensors on their clothing, and video recordings were compared to sensor data. Bluetooth data reflected general direction of movement and instances of idling but were neither precise or accurate. RFID data was accurate after applying data filters. Both systems detected multiple sensors simultaneously. The Bluetooth system is not feasible for clinical use, but the RFID system shows potential for clinical application and accurate measurement of social network factors as interaction frequency and duration.

